# Same Data; Different Interpretations

**DOI:** 10.1200/JCO.2016.68.2021

**Published:** 2016-08-29

**Authors:** Bishal Gyawali, Vinay Prasad

**Affiliations:** Bishal Gyawali, Nobel Hospital, Sinamangal, Kathmandu, Nepal; Vinay Prasad, Oregon Health and Sciences University, Portland, OR

Interpretation of oncology clinical trial data are not always straightforward or consistent. Similar trial results with disparate interventions may be interpreted differently by the oncology community. One of the main reasons for this discrepancy is the debate regarding what is the appropriate end point for demonstration of efficacy of cancer drugs. There is no doubt that overall survival (OS) is the best parameter to judge the utility of any intervention, and it is free from bias in ascertainment and measurement^1^; but for conditions with few treatment options and dire outcomes, the need for new agents is high and the oncology community sometimes settles on a surrogate end point that, in many cases, is progression-free survival (PFS).^2^ It is easy to understand why PFS is favored among the researchers: It occurs early and is not influenced by postprogression therapy. At the same time, it would make little sense to have an agent that reduces chances of dying of cancer but increases off-target deaths; hence, the need for verification of OS. Phase III trials that report on significant PFS benefits without OS prolongation become the apples of discord in the oncology community. In this commentary, we present three examples from lung, ovarian, and breast cancers and demonstrate how the oncology community interprets similar data differently. Finally, we take our best guess as to why this phenomenon happens.

## Lung Cancer: Bevacizumab and Cetuximab

Bevacizumab and cetuximab have both been tested in phase III trials for use in advanced/metastatic non-small cell lung cancer (NSCLC) in combination with chemotherapy. The Eastern Cooperative Oncology Group (ECOG) 4599 trial demonstrated a significant OS prolongation with the addition of bevacizumab compared with chemotherapy alone (12.3 months *v* 10.3 months; hazard ratio [HR], 0.79; *P* = .03) but with significant toxicities, including 15 treatment-related deaths among 434 patients randomly assigned to the bevacizumab arm.^3^ The AVAIL (Avastin in Lung) study on the other hand found a marginal benefit in PFS, with no benefit in OS, by adding bevacizumab to chemotherapy (13.6 months *v* 13.1 months; HR, 0.93; *P* = not significant [NS]).^4^ A Japanese study also failed to show an OS benefit with addition of bevacizumab to chemotherapy (22.8 months *v* 23.4 months; HR, 0.99; *P* = .95).^5^ However, bevacizumab received approval by the US Food and Drug Administration (FDA) for use in this setting and is commonly used in practice as evidenced by its inclusion in the National Comprehensive Cancer Network (NCCN) guidelines as a category 2A recommendation for patients with EGFR, ALK negative, or unknown nonsquamous non-small cell lung cancer.^6^

FLEX (First-Line Erbitux in Lung Cancer) was a randomized phase III trial comparing chemotherapy plus cetuximab with chemotherapy alone in patients with advanced NSCLC and demonstrated a significant OS benefit (11.3 months *v* 10.1 months; HR, 0.87; *P* = .044).^7^ However, another phase III trial, BMS099, failed to show similar benefit in OS (9.6 months *v* 8.3 months; HR, 0.89; *P* = .169).^8^ It is important to note here that OS was the primary end point in FLEX, whereas PFS was the primary end point in the BMS099 study. Later, a meta-analysis showed significant benefit for OS, PFS, and response rates with the addition of cetuximab to chemotherapy.^9^ However, cetuximab is not approved by the FDA and is widely considered a failed drug in NSCLC by the oncology community, as evidenced by its removal from the NCCN guidelines.^6^

## Ovarian Cancer: Angiogenesis Inhibitors and Dose-Dense Chemotherapy

Several attempts have been made to build on the success of the platinum-taxane combination for treating advanced or metastatic ovarian cancer, but none have been met with irrefutable success. Of those various strategies, two are the most common and the most debated: dose-dense treatment schedule and addition of an angiogenesis inhibitor to the combination.

The feasibility and efficacy of a dose-dense schedule (weekly paclitaxel *v* every-3-week paclitaxel) was demonstrated in the Japanese Gynecologic Oncology Group (JGOG) 3016 trial, a study among 637 Japanese patients.^10^ This trial showed that weekly paclitaxel improved both PFS and OS. The OS advantage was not trivial; it was a sizable 38-month extension (100.5 months *v* 62.2 months; HR, 0.79; *P* = .039). However, the global oncology community adopted the addition of bevacizumab but has largely ignored the dose-dense paclitaxel schedule. Perhaps, the large benefit with weekly paclitaxel prompted clinicians to disbelief and wanting further confirmation; yet, it is hard to imagine clinicians believed a larger benefit would altogether vanish, rather than merely be attenuated.

In 2014, an Italian trial failed to replicate these results, but had used a different dose schedule.^11^ Whether this lack of replication was due to this difference in dose of paclitaxel used or due to ethnic differences between the populations remains to be known, but the results of the Gynecologic Oncology Group (GOG-0262) trial have shown benefit with weekly paclitaxel in the US population.^12^

In the past few months, three important clinical trials have been published and add to the evidence (and confusion) of these two strategies: the updated results of the International Collaborative Ovarian Neoplasm 7 (ICON7) trial,^13^ the AGO-OVAR 12 (Standard first-line chemotherapy with or without nintedanib for advanced ovarian cancer) trial,^14^ and the GOG-0262 trial.^12^ The results of these trials and the conclusions the authors derived are of interest and importance.

The ICON-7 trial showed a PFS benefit but failed to show an OS benefit with the addition of bevacizumab to the chemotherapy backbone.^13^ However, a subgroup analysis was performed and revealed that for high-risk patients, addition of bevacizumab did have an OS benefit. Instead of highlighting the overall negative OS data, the authors chose to emphasize the OS advantage among high-risk patients. Further, this trial was not placebo controlled and has been criticized.^15^

The AGO-OVAR 12 trial randomized a large number of patients (N = 1,366) to nintedanib, another angiogenesis inhibitor, or placebo in combination with chemotherapy.^14^ OS data are not available but PFS was significantly better with nintedanib versus placebo (HR, 0.84; *P* = .024). However, the actual gain in PFS was a mere 0.6 months. But the authors concluded, “Nintedanib in combination with carboplatin and paclitaxel is an active first-line treatment that significantly increases progression-free survival for women with advanced ovarian cancer.”^14^

GOG 0262, the third study, compared weekly paclitaxel with every-3-week paclitaxel among patients with ovarian cancer.^12^ This trial also allowed patients to receive bevacizumab and prospectively stratified them according to bevacizumab status. Although every-3-week paclitaxel did not improve the PFS in the entire population (14.7 months *v* 14.0 months; HR, 0.89, *P* = .18), the PFS for those patients who did not take bevacizumab was significantly improved by 3.9 months (14.2 months *v* 10.3 months; HR, 0.62; *P* = .03).^12^ The OS data are not yet available. Considering that 84% of patients in this trial took bevacizumab and this could negate the overall benefit of dose-dense treatment, we assumed the trial would be interpreted as meaning weekly paclitaxel was superior to every-3-week dosing except for those patients who received additional bevacizumab. However, the results of this trial have mostly been interpreted as negative.

Important information can be gleaned from summarizing these trials. There is no trial that shows OS benefit with any angiogenesis inhibitor in ovarian cancer (Table 1), whereas there is one trial that shows OS benefit with the dose-dense schedule. Unless we have data comparing chemotherapy plus bevacizumab versus dose-dense chemotherapy alone, the current evidence equally (if not more) supports the use of dose-dense chemotherapy alone compared with bevacizumab addition.

**Table 1.**
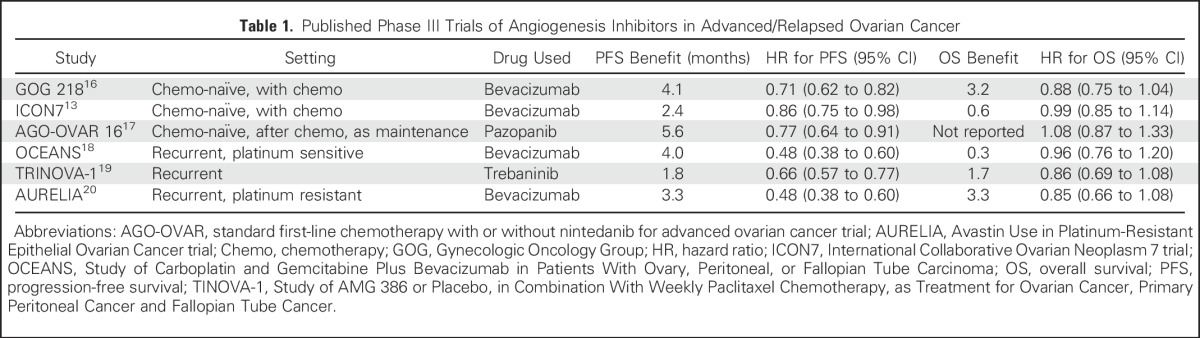
Published Phase III Trials of Angiogenesis Inhibitors in Advanced/Relapsed Ovarian Cancer

Yet, the authors of such pivotal studies as ICON7 attempted to emphasize the benefit of bevacizumab, whereas those of GOG-0262 did not highlight the benefits derived from dose-dense paclitaxel. It is noteworthy that practice patterns occur despite the fact that the NCCN guidelines categorize bevacizumab addition to first-line chemotherapy as a category 3 recommendation and dose-dense paclitaxel as a category 1.^21^

## Breast Cancer: Everolimus and Bevacizumab

The use of PFS as a surrogate for OS may be valid for certain tumor types, certain classes of agents, and certain lines of therapy, but an umbrella analysis of surrogate correlation studies showed that it is unreliable in the setting of metastatic breast cancer.^1^ This uncertainty took on importance after the results of the E2100 trial, which showed a large improvement in PFS from the use of bevacizumab when added to taxane therapy versus taxane therapy alone.^22^ This finding led to accelerated approval of the drug. Yet, just a few years later, multiple randomized trials not only failed to confirm survival benefit in this setting but also failed to replicate similar magnitude of benefit in PFS. And, bevacizumab clearly increased toxicity. After a contentious fight, the drug was revoked.

Now, just 4 years later, we have seen two new drug approvals for metastatic breast cancer that mirror the history of bevacizumab. Everolimus^23^ and palbociclib,^24^ both in combination with hormonal therapy, have had markedly similar results to the case of bevacizumab. Both drugs improved PFS in randomized trials, both drugs add toxicity, and neither drug has shown OS benefits. It is interesting to note that the absolute gain in PFS in the pivotal trials of these drugs is similar to that of bevacizumab seen in the E2100 trial: 4.1 months with everolimus,^23^ 5.4 months with palbociclib,^24^ and 5.9 months with bevacizumab.^22^ However, a meta-analysis conducted later, with the addition of subsequent trials, showed that the pooled benefit in PFS with bevacizumab was only 2.5 months.^25^ Whether the PFS benefits with everolimus and palbociclib also are similarly reduced remains to be seen with the acquisition of more data. At least in the case of everolimus, the drug received traditional or full approval, meaning that revoking the approval on the basis of further efficacy data is unlikely, and postmarketing studies to assess the drug’s benefit on OS are not required. Still, the NCCN guidelines include both bevacizumab and everolimus as a category 2A recommendation, whereas palbociclib gets a category 1 recommendation—without having any OS data yet!^26^

## Same Data; Different Interpretations

It is difficult to provide a unifying theme that explains why we treat similar data differently in oncology. Potential explanations include reimbursement incentives, historical accident, pharmaceutical marketing, perceived toxicity, clinical anecdotes, social norms, or objective and articulable differences that we have not considered. In the case of bevacizumab and cetuximab in NSCLC, the unique regulatory history and pathway for drug approval likely explain the success and validation of the former and the failure of the latter. In the case of discrepancy among the oncologists in the acceptance of dose-dense chemotherapy versus angiogenesis inhibitors in ovarian cancer, it is difficult to not consider the issue of financial reimbursement (higher with bevacizumab) and convenience to practitioners.

A more optimistic outlook of the medical community toward targeted therapies compared with cytotoxic agents may be another potential reason. In the final case of everolimus and bevacizumab, it is possible regulators were not eager to relive the painful events leading to removal of bevacizumab’s indication, and, for that reason, gave an unwarranted traditional (full) approval to everolimus (on the basis of comparable data). This would eliminate the need for postmarketing studies and preclude a contentious withdrawal from market, as was seen for bevacizumab. Ultimately, however, our interpretation of these discrepancies must be acknowledged as speculative and other potential factors in play for these discrepancies must be explored. Given that we now have umbrella meta-analyses of the strength of surrogate correlations in oncology^1,27^ that show the validity of correlations between surrogates and survival in specific cancer settings (e.g., does disease-free survival predict OS among cytotoxic drugs in the adjuvant treatment of colorectal cancer?), it may now be possible for the field to move toward greater evidence-based consistency in our interpretation and regulatory use of trial data.

We cannot also ignore the deep issues beyond clinical data that result in discrepancies in cancer care, such as politics, emotional overlay, lobbying, and advocacy of support groups. Although we explore three instances of discrepancies in the treatment of three similar cancer settings in this paper, many discrepancies exist in cancer care. When bevacizumab was revoked for breast cancer, support groups and patient advocates protested against the decision, but when ^131^I-tositumomab was withdrawn from marketing, it died silently. Thus, our attitudes toward cancer care are multifactorial. As oncologists, however, we should push for uniformity in the interpretation of clinical trial results and try to achieve as much consistency in our practice as possible. Consistency would be a virtue for cancer care.
